# Congenital Vocal Cord Paralysis and Late-Onset Limb-Girdle Weakness in MuSK–Congenital Myasthenic Syndrome

**DOI:** 10.3389/fneur.2019.01300

**Published:** 2019-12-20

**Authors:** Marcus V. Pinto, Jacqui-Lyn Saw, Margherita Milone

**Affiliations:** Department of Neurology, Mayo Clinic, Rochester, MN, United States

**Keywords:** congenital myasthenic syndrome, muscle skeletal receptor tyrosine kinase, limb girdle, vocal cord paralysis, CMS, MuSK, Neuromuscular Junction disorder

## Abstract

A 30-year-old woman with congenital vocal cord paralysis presented for evaluation of fatigable proximal upper limb weakness and difficulty maintaining the neck erect. Neurologic examination showed bilateral asymmetric eyelid ptosis, mild weakness (MRC 4/5), and atrophy of neck extensors and shoulder girdle muscles, whereas lower limb muscle strength was normal. Repetitive nerve stimulation revealed decremental responses in orbicularis oculis and trapezius. Needle electromyography demonstrated myopathic changes in proximal and paraspinal muscles. Acetylcholine receptor and muscle skeletal receptor tyrosine kinase (MuSK) antibodies, creatine kinase (CK), and lactate were negative or normal. Next-generation sequencing detected two heterozygous variants in the *MUSK* gene. One variant, c.79+2T>G, is a known pathogenic variant, and the other, c.2165T>C (p.V722A), is a novel missense variant, predicted to be pathogenic by *in silico* analysis. The two variants were proven to be in *trans*. This case expands the clinical and molecular spectrum of MuSK congenital myasthenic syndromes.

## Introduction

Congenital myasthenic syndromes (CMS) are a heterogeneous group of hereditary neuromuscular disorders due to defects of the neuromuscular transmission. CMSs are characterized by fatigable muscle weakness, ptosis, and ophthalmoparesis and often manifest in infancy or childhood (1). However, a fair number of CMS patients remain undiagnosed until adulthood. Mutations in at least 30 genes encoding for proteins involved in the neuromuscular transmission have been linked to CMS (2). Muscle skeletal receptor tyrosine kinase (MuSK) CMS is a rare autosomal recessive CMS, and only 22 cases have been reported worldwide (3–13). MuSK is a post-synaptic transmembrane protein consisting of three immunoglobulin-like domains, a frizzled cysteine-rich domain, and a protein kinase domain. It has a crucial role in the formation and stabilization of the neuromuscular junction (14), and its disruption in mice has been shown to result in failure to form neuromuscular junctions and death (15). MuSK deficiency usually presents at birth with eyelid ptosis and/or respiratory distress, followed by ocular, facial, and proximal limb muscle weakness (1). However, it can a wider phenotypic spectrum that includes fetal akinesia deformation sequence syndrome (16), congenital vocal cord paralysis (CVCP) (13), or late-onset limb-girdle weakness (12). CVCP was previously reported as presenting symptom of Dok7 CMS, rare cases of fast-channel syndrome, and later in MuSK deficiency CMS (1, 9, 13). However, all patients with MuSK CMS and CVCP at disease-onset developed weakness in childhood or presented with isolated CVCP (9, 10, 12, 13). Herein, we describe a patient with MuSK CMS manifesting with CVCP and late-onset limb-girdle weakness.

## Case Report

**Figure 1:**
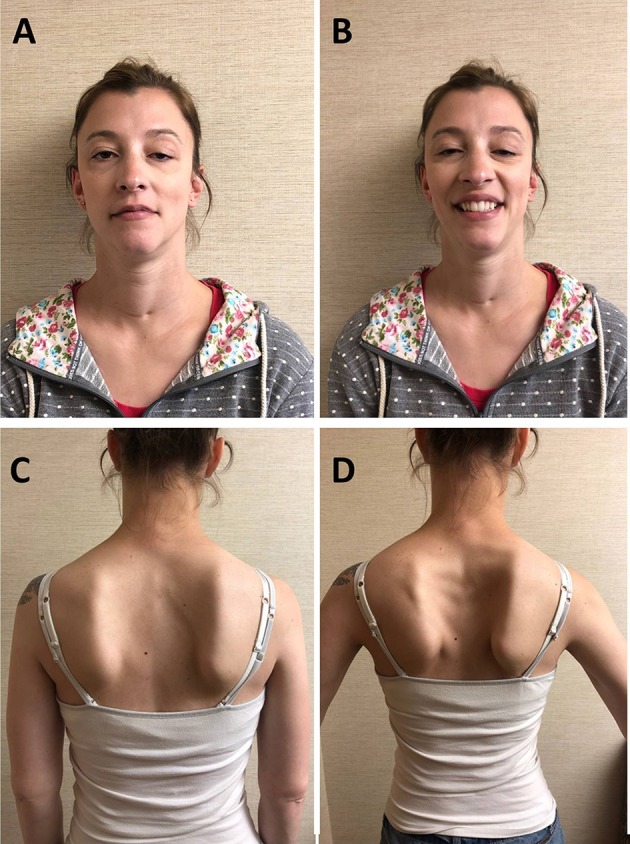
Patient. **(A)** Bilateral asymmetric ptosis. **(B)** Limited smile due to facial weakness, micrognathia, and long neck; scar at base of neck due to tracheostomy in infancy. **(C)** Atrophy of the shoulder girdle and thoracic paraspinal muscles. **(D)** Bilateral scapular winging.

The patient is a 30-year-old woman who was born at term to non-consanguineous parents. Immediately after uncomplicated delivery, she developed respiratory distress and required intubation. She received a diagnosis of bilateral CVCP. She was discharged home with a tracheostomy without ventilator support or oxygen. She had no episodes of apnea. She underwent bilateral arytenoidectomy was successfully decannulated at age 3. In the mean time, she achieved normal motor milestones and kept up with her peers in gymnastics. She had, however, some exertional dyspnea and stridor. Mild eyelid ptosis was noted in childhood. During pregnancy, 15 months before presenting to our institution, she noted fatigable weakness of proximal upper limb and neck extensors muscles as well as shoulder girdle muscle atrophy. She had no diplopia, dysphagia, lower limb weakness, or exacerbation of the respiratory symptoms. She had no myalgia. Her 11-month-old daughter is well and has met motor development on time. Her 33-year-old brother and parents, of English and German descent, are asymptomatic. Neurological examination showed bilateral asymmetric eyelid ptosis (Figures 1A,B), slight ophthalmoparesis without diplopia, moderate lower facial weakness, mild tongue weakness, and mildly reduced elevation of the soft palate. Neck extensors and shoulder girdle muscles were mild weak and atrophic [Medical Research Council (MRC) 4/5], whereas lower limb muscle strength was normal. Deltoid fatigability was observed. She had mild high arched palate, micrognathia, long neck, bilateral scapular winging (Figures 1C,D), and hyperextensibility of the interphalangeal and metacarpophalangeal joints. Repetitive nerve stimulation at 2 Hz revealed decremental responses in orbicularis oculis (−7% at rest and −15% post-exertion) and trapezius (−22% at rest and −35% post-exertion). The more pronounced decrement in the trapezius, compared with that recorded in the orbicularis oculi, reflected the clinically more prominent involvement of the shoulder girdle muscles compared with the relatively spared orbicularis oculi. There was no facilitation. Needle electromyography demonstrated myopathic changes without abnormal spontaneous muscle activity in proximal and paraspinal muscles. Acetylcholine receptor and MuSK antibodies, creatine kinase (CK), and lactate were negative or normal. Pulmonary function tests showed reduced maximal respiratory pressures with features suggestive of fixed central airway obstruction. Next-generation sequencing detected two heterozygous variants in the *MUSK* gene. One variant, c.79+2T>G, affects a donor splice site in intron 1 and is expected to result in absent or disrupted protein product. This variant was previously reported in two MuSK CMS patients with CVCP (9). The second variant, c.2165T>C (p.V722A), is a novel missense variant that affects a highly conserved amino acid, not present in the population databases and predicted to be deleterious by SIFT and PolyPhen-2 P2. The two variants were proven to be in *trans* (C.79+2T>G from mother and c.2165T>C from father). Albuterol 4 mg orally resulted in normal strength of the neck flexors, neck extensors, biceps brachii, and triceps bilaterally.

## Discussion

This patient expands the clinical and molecular spectrum of MuSK CMS, a rare form of neuromuscular junction defect. Indeed, she presented with adult-onset limb-girdle weakness, in addition to CVCP, and compound heterozygosity for a novel missense *MUSK* variant. The novel p.V722A variant is located in the tyrosine kinase region of MuSK and very close to a previously reported pathogenic variant (p.A727V) (12). Owen et al. described four MuSK CMS patients with late-onset limb-girdle predominant weakness and eyelid ptosis who were diagnosed in adulthood, but none of them had CVCP. These adult patients carried compound heterozygous *MUSK* variants affecting exclusively the protein kinase region, leading the authors to hypothesize that mutations in this region may be specific for late-onset limb-girdle weakness (12). However, recently, isolated CVCP was reported in two children (siblings) with compound heterozygous variants in the tyrosine kinase domain of MuSK (p.A763T and p.R816X) (13). Dok7 CMS is one of the most common CMS diagnosed in adulthood and often results in a mild phenotype with limb-girdle predominant weakness (2), as observed in our patient, mimicking a limb-girdle muscular dystrophy. It is unknown why our patient, like other CMS patients (1, 2), had initial symptoms very early in life and then partially improved but developed limb-girdle weakness decades later. Similarly to MuSK CMS, also Dok7 CMS can cause CVCP and usually spares extra-ocular muscles (1, 5, 12, 13). The phenotypic overlapping between these two forms of CMS could stem from a similar underlying mechanism, secondary to the close interplay between MuSK and Dok7. MuSK is activated extracellularly by the neural isoform of agrin through binding LDL receptor related protein 4 (LRP4) and intracellularly by Dok7 (1, 5, 15). Dok7 recruits MuSK through phosphorylations of its Tyr553 residue, which then leads to phosphorylation of the acetylcholine receptor (AChR) β-subunit, AChR clustering, and reorganization of the actin cytoskeleton (1, 5). Severe impairment of the interaction between MuSK and Dok7, resulting in abnormal endplate formation and presynaptic differentiation, was demonstrated in a compound heterozygous MuSK CMS patient (5). Mice lacking MuSK do not develop neuromuscular junctions and die at birth from respiratory failure, confirming the vital role of the MuSK signaling pathways (15).

Our patient, similarly to most MuSK CMS patients (12), responded to albuterol, which should be the first-line therapy in MuSK CMS and other CMS featured by impairment of the agrin-LRP4-MuSK-Dok7 pathway. Acetylcholinesterase inhibitors may worsen weakness in MuSK CMS, as in MuSK-antibody positive myasthenia gravis, and should be avoided.

## Ethics Statement

Written informed consent was obtained from the participant for the publication of this case report and all identifiable data and images.

## Author Contributions

MP and MM performed data collection, drafting of final manuscript, approval, and critical review of final form. J-LS performed data collection, approval, and critical review of final form.

### Conflict of Interest

MM receives compensation to serve as associate editor of *Neurology Genetics*. The remaining authors declare that the research was conducted in the absence of any commercial or financial relationships that could be construed as a potential conflict of interest.

## Abbreviations

CMS: congenital myasthenic syndromes
CVCP: congenital vocal cord paralysis
MuSK: muscle skeletal receptor tyrosine kinase
*MUSK*: muscle skeletal receptor tyrosine kinase gene.
